# Sequential Dynamics of Stearoyl-CoA Desaturase-1(SCD1)/Ligand Binding and Unbinding Mechanism: A Computational Study

**DOI:** 10.3390/biom11101435

**Published:** 2021-09-30

**Authors:** Anna B. Petroff, Rebecca L. Weir, Charles R. Yates, Joseph D. Ng, Jerome Baudry

**Affiliations:** 1 Department of Biological Sciences, The University of Alabama in Huntsville, Huntsville, AL 35899, USA; anna.petroff@uah.edu (A.B.P.); ngj@uah.edu (J.D.N.); 2 Department of Biochemistry and Cellular and Molecular Biology, The University of Tennessee, Knoxville, TN 37996, USA; rebeccaweir12@gmail.com; 3 National Center for Natural Products Research, The University of Mississippi School of Pharmacy, Oxford, MS 38677, USA; cryates2@olemiss.edu

**Keywords:** stearoyl-CoA desaturase-1, delta-9 desaturase, membrane protein, molecular dynamics, desaturase, molecular modeling

## Abstract

Stearoyl-CoA desaturase-1 (SCD1 or delta-9 desaturase, D9D) is a key metabolic protein that modulates cellular inflammation and stress, but overactivity of SCD1 is associated with diseases, including cancer and metabolic syndrome. This transmembrane endoplasmic reticulum protein converts saturated fatty acids into monounsaturated fatty acids, primarily stearoyl-CoA into oleoyl-CoA, which are critical products for energy metabolism and membrane composition. The present computational molecular dynamics study characterizes the molecular dynamics of SCD1 with substrate, product, and as an apoprotein. The modeling of SCD1:fatty acid interactions suggests that: (1) SCD1:CoA moiety interactions open the substrate-binding tunnel, (2) SCD1 stabilizes a substrate conformation favorable for desaturation, and (3) SCD1:product interactions result in an opening of the tunnel, possibly allowing product exit into the surrounding membrane. Together, these results describe a highly dynamic series of SCD1 conformations resulting from the enzyme:cofactor:substrate interplay that inform drug-discovery efforts.

## 1. Introduction

**Figure 1:**
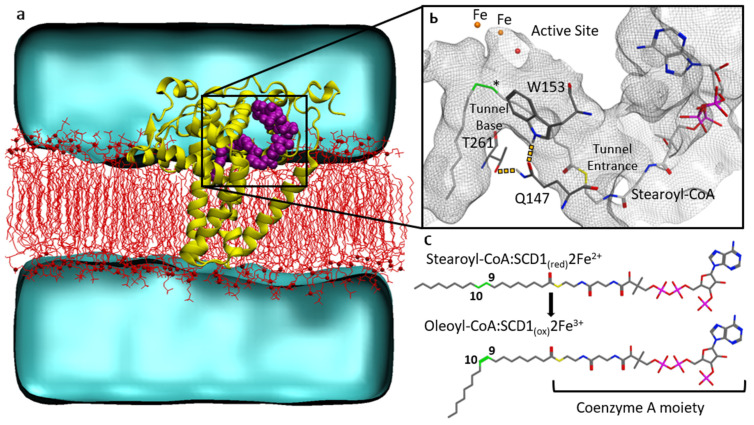
Stearoyl-CoA desaturase 1 Δ-9 desaturation reaction: (**a**) SCD1 desaturase (yellow) in POPC membrane (lipids as red lines, phosphate groups as red spheres), with stearoyl-CoA substrate (purple), water environment (cyan); (**b**) SCD1 hydrophobic tunnel (gray mesh) with stearoyl-CoA (colored by atom type). Substrate 9th and 10th carbons (green) positioned proximal to the active site’s diiron center (orange), and the oxygen atom of crystallographically determined water (red). Hydrogen bonding between residues in yellow dashes. (**c**) The SCD1 desaturation reaction, resulting in a double bond at the Δ-9 position (indicated with asterisk), converting stearoyl-CoA (substrate) to oleoyl-CoA (product).

Stearoyl-CoA desaturase-1 (SCD1), an endoplasmic reticulum membrane enzyme, is a central regulator of energy metabolism [1]. SCD1 desaturates stearoyl-CoA and palmitoyl-CoA into the monounsaturated fatty acids (MUFA) oleoyl-CoA and palmitoleoyl-CoA through the insertion of a double bond in the Δ-9 position of the substrate [2] (Figure 1a–c), as indicated by SCD1’s alternative name, delta-9 desaturase (D9D). This oxidative reaction requires electron transport cytochrome b5 and molecular oxygen [2]. The well-controlled activity of SCD1 is critically important to health as the products are used in the formation of phospholipids, triglycerides, and cholesteryl esters, and hence contribute to membrane fluidity, adiposity, and signal transduction [3]. SCD1 is ubiquitously expressed, particularly in lipogenic tissues, and its concentration changes in response to hormonal and dietary effectors [4].

While SCD1 activity is critical to health, particularly because it is important to the modulation of cellular inflammation and stress [5], SCD1 activity can be associated with disease under certain conditions. Under high-fat conditions, SCD1 deficiency protects mice from obesity, insulin resistance [6], and hepatic steatosis [7]. Similarly, high SCD1 activity predicted the development of metabolic syndrome in men [8]. SCD1 overexpression is implicated in cancer [9,10], and it has been described as an oncogene [11]. As such, SCD1 is a potentially important pharmaceutical target; inhibitors of SCD1 have been developed as potential treatments for diabetes [12] and cancer [13,14]. Because SCD1 activity has nutritional effectors, nutraceuticals have been investigated as mitigators of SCD1 overactivity. For example, Manni et al. [15] found that treatment with the drug Lovaza^®^, made of ethyl esters of the omega-3 fatty acids DHA and EPA, resulted in lower levels of SCD1 products in obese postmenopausal women at high risk for breast cancer. In addition, results from a mouse model of alcoholic liver disease indicate that the naturally occurring SCD1 inhibitor *Lycium barbarum* polysaccharide, has liver-protective effects [16]. Nonetheless, the development of pharmaceutical or dietary modulators of SCD1 has been challenging because of variations in expression by organ type [17].

The recent determination of human [18] and murine [19] SCD1 crystal structures provides fundamental insights into the mechanism of action of the enzyme and of its specificity. These findings open the door to a rational, structure-based modulation of its activity. Both human and murine SCD1 structures exhibit a bent, or kinked, binding site for the saturated-CoA substrate. In their murine model, Bai et al. [19] attribute the kink to the hydrogen bond interactions between three residues that underlie the binding tunnel. The homologous human protein structure [18] reveals the same interaction pattern between these conserved residues; Gln147 interacts with both Trp153 and Thr261 (Figure 1b). Bai et al. [19] suggest that this binding site geometry drives the conformation of the saturated substrate acyl chain such that the regioselective desaturation can occur at C9, as was first hypothesized in 1969 [20]. Inhibition and analog studies [2] indicate that the carbons C9 and C10 of the substrate’s lipid tail must adopt a negative gauche conformation, −60° dihedral, in order for the rate-limiting hydrogen abstraction that results in the cis double bond to occur. However, the crystal structure of human SCD1 exhibits a non-gauche conformation with a −111.1° C8-C9-C10-C11 dihedral angle [18]. Because the crystal structure determined a C9 and C10 dihedral other than the actionable −60° dihedral, it is unclear how the hydrogen bonding interactions between the two residue pairs (i.e., Gln147-Trp153 and Gln147-Thr261) impact the probability of the substrate dihedral to favor an actionable conformation for the desaturation to occur. The human and mouse SCD1 crystal structures also exhibit several non-bonded contacts between the CoA moiety of the substrate and the protein’s cytoplasmic domains, suggesting a possible active effect of CoA in the positioning of substrates, and are consistent with assay results indicating that the CoA moiety is required for binding [2].

SCD1’s human disease relevance, including cancer and metabolic syndrome, makes it an important candidate disease target. However, although there are numerous studies concerning scd1 expression, genetic variation, and transcriptional regulation [3,4,5,7,21,22,23], the only known mutagenesis studies on the SCD1 protein concern either the residues that contribute to catalysis [24] or the residues that may be mutated to prevent the quick degradation of this short-lived membrane protein [25]. Mutagenesis results from other desaturases, such as those that desaturate a different carbon-carbon bond (e.g., delta-6 rather than delta-9), interact with a different headgroup (e.g., the acyl carrier protein rather than CoA), or occupy a different area of the cell (e.g., solvated vs. membrane), do not provide results that can be extrapolated to inform the workings of SCD1. For instance, rat delta-6 desaturase [26] is also a membrane protein that desaturates lipids with CoA headgroups. While mutagenesis studies indicate that the binding tunnel has residues that contribute to substrate selection [26], the rat D6D-human D9D identity is only 21%. Furthermore, it is not known if rat D6D has a similar structure to human D9D despite this difference in identity. Vanhercke et al. [27], who published mutagenesis results on a bifunctional Δ-12/Δ-9 membrane-bound desaturase found in crickets, state that due to the lack of structural data on membrane proteins, “our understanding of the structure-function relationship of membrane-bound desaturases remains limited and scattered at best” (p. 12860). Similar efforts to characterize catalysis on fatty acids face the same problem; even when site-directed mutagenesis studies exist, without structural data, interpretation is limited [28]. The recently determined structures of SCD1 [18,19] open the door to a much-needed functional characterization of the ligand:protein interactions beyond the catalytic mechanism.

A better understanding of the substrate-protein interaction in the binding site dynamics of SCD1 and its substrates is needed. Hence, results of the present work help to guide and prioritize future mutagenesis wet-lab experiments. The recently determined crystal structures identified three residues thought to confer a kinked binding tunnel that facilitates substrate position for desaturation. However, it is unknown from the crystal structures to what extent the hydrogen bonding network between these residues can lead to an actionable dihedral at C9-C10, or if the hydrogen bonding pattern is fixed or changes upon different bound species (i.e., substrate or product).

To answer these questions, the present work characterizes SCD1’s conformational dynamics when the substrate is present, as well as the dynamics across the protein turnover cycle: apoprotein to protein–substrate to protein–product, beginning again as apoprotein. These provide a description that is contrasted with the dynamics of the SCD1:substrate model built from the crystal structure.

## 2. Materials and Methods

### 2.1. Model Construction and Simulations

All models (described in Table 1) are based on PDB entry 4ZYO, the human SCD1 structure determined by Wang et al. [18]. In building the transmembrane SCD1 model, the enzyme and substrate atoms were included in the model. The co-crystalized dodecyl-beta-D-maltoside was deleted, and the zinc ions in the crystal structures were replaced by the naturally occurring iron ions at the same locations. The force field parameters for stearoyl-CoA were generated with CHARMM General Force Field (CGenFF) [29], and using the CHARMM36 [30] force field for the protein, lipid, water, and ion parts of the model. Membrane generation, placement, and preparatory steps were performed using the QwikMD [31] and NAMD2 [32,33] programs, and the SHAKE algorithm [34,35,36]. From an initial orientation obtained using the OPM server [37], the protein was placed into a model membrane made of 1-palmitoyl-2-oleoyl-sn-glycero-3-phosphocholine (POPC), the most common ER membrane lipid species [38]. The protein and membrane were solvated with TIP3P water models with a salt concentration of 0.15 mol/L of NaCl in a periodic box of 100 Å × 100 Å × 100 Å, representing a total of 95,297 atoms.

The membrane-protein model was submitted to the following procedure, all using explicit hydration, with a 12 Å non-bonded interactions cutoff, and using particle mesh Ewald. First, the heavy atoms of the protein backbone, the active site residues (HIS120, HIS125, HIS127, HIS160, HIS161, ASN265, HIS269, HIS298, HIS301, HIS302), and the stearoyl-CoA ligand, were restrained using a harmonic restraint of 2 kcal/mol Å^2^ about their crystallographic positions. Next, the membrane lipids were annealed to the protein (i.e., allowed to surround the protein as they would in a biological membrane) over the course of 30 ns with the same active site restraints using the QwikMD Toolkit in VMD. The model system was then equilibrated for 1 ns with the same restraints on the atomic positions. Finally, an additional 1-ns molecular dynamics (MD) simulation equilibration was run with the stearoyl-CoA acyl tail carbons restrained and the CoA unrestrained. The resultant model system contained no water molecules inside the membrane. The equilibrated model is shown in Figure 1a. As shown, the protein structure was oriented such that the four transmembrane helices extend across the membrane, with amphipathic helices at the membrane–solvent interface, and a protein cap with the active site extending into the cytosol (Figure 1a). This position is consistent with that described in Wang et al. [18]. The average membrane thickness around the protein during the MD simulation was 32.1 ± 0.2 Å.

Using this equilibrated model, a series of different models were built, with each differing from each other by the type of ligand present. These modified ligands, shown in Table 1, were constructed using the program MOE version 2019 (Chemical Computing Group, Montreal, QC, Canada), which generates amber 99 force-field parameters for each ligand. For each variant, the solvent was first deleted from the membrane–protein system. The desired ligand was included in the model, and the system was re-solvated using the MOE solvate facility 0.15 mol/L Na^+^Cl^−^ counterions. The distances between the iron ions and the coordinating histidine or water residues were restrained around their crystallographic positions using harmonic potentials of 40 kcal/mol Å^2^. Each model was included in a 100 Å × 100 Å × 100 Å periodic box, which totaled between 99,577 and 100,161 atoms, depending on the model. The models are: (1) model “Substrate”: stearoyl-CoA, i.e., with a lipid fatty acid chain with a C9-C10 single bond, (Figure 1c) in SCD1, as shown in Table 1a; (2) model “Product”: oleoyl-CoA, i.e., with a lipid fatty acid chain with a C9-C10 double bond, in SCD1, as shown in Table 1b; (3) model “Apoprotein”: SCD1 without substrate, as in Table 1c; (4) model “CoA”: CoA moiety, without the fatty acid, in SCD1, as shown in Table 1d. In order to account for the protein tunnel’s contribution to the ligand shape, two additional models were built containing only ligands in a waterbox (i.e., containing no protein and no membrane): (5) model “Substrate-waterbox”: stearoyl-CoA (i.e., as in Table 1a) in a periodic water box 40 Å × 40 Å × 40 Å, as shown in Table 1e.

Each model was energy-minimized to an energy gradient of less than 10^−4^ kcal/mol/Å^2^ followed by a 1-ns MD equilibration simulation and a 100-ns production run Each of the models was repeated five times, resulting in a combined production simulation time of 500 ns for each model, two microseconds in total.

### 2.2. Molecular Dynamics Analysis

MD trajectory measurements were used to measure the dihedral angle values of C8, C9, C10, and C11, as well as the distance between the two irons of the active site and the distance from each iron to C9 and C10. Both human and murine crystal structures indicated a kinked binding tunnel. Bai et al. [19] proposed that the tunnel kink is due to hydrogen bonding between Gln147, Trp153, and Thr261. The present work defined these distances as follows: (1) the distance between Gln147 (Oε1) and Trp153 (hydrogen of indole nitrogen Hε1), and (2) the distance between Gln147 (Hε22) and Thr261 (Oγ1), as shown in Figure 1b. In the human crystal structure [18], the 5th carbon of the stearoyl-CoA lipid tail appears at the tunnel entrance. The overall structure was solved at 3.25 Å resolution. Several residues close to the residues of interest listed here exhibited relatively high B factors: Trp153 (atoms C, CD2, and CE3, with B factors of 101.69, 101.14, and 100.12, respectively), and Thr143 (atom OG1 with a B factor of 106.69). Volume measurements were centered on this 5th carbon and extend at a 6 Å radius, resulting in a sphere that includes the three residues of interest. Volume measurements were obtained using the program POVME3 [39] from the five 100-ns MD trajectories.

## 3. Results

### 3.1. Structure and Dynmaics of Substrate, Product, Apoprotein, and CoA Models

Comparison of the combined Substrate, Product, Apoprotein, and CoA models (Table 1a–d, respectively) indicates that the Substrate model maintained a stable, close proximity between the Gln147, Trp153, and the Thr261 residues (Table S1), suggested to be responsible for the kinked conformation of the binding site [19]. The Product, Apoprotein, and CoA models exhibited greater and more varied distances, particularly between Gln147 and Thr261, which is the interaction that extends across the base of the tunnel (Figure 1b). The Gln147-Trp153 distance, which is alongside the tunnel, was within 2.0 Å to 2.5 Å across the model and simulations.

The distance between the metal ions in the crystal structure of human SCD1 is 6.8 Å [18]. During the molecular dynamics simulations, that same distance was found to vary between 6.5 Å and 10.5 Å, with the most frequently sampled distances between 7.5 Å to 7.8 Å (Figure S1). This finding indicates that while the molecular dynamics simulation and the removal of the crystal packing may increase the iron–iron distance compared to the static crystal structure, the structure of the protein active site is maintained overall.

### 3.2. Substrate Tunnel Kink and Protein’s Hydrogen Bond Network

**Figure 2:**
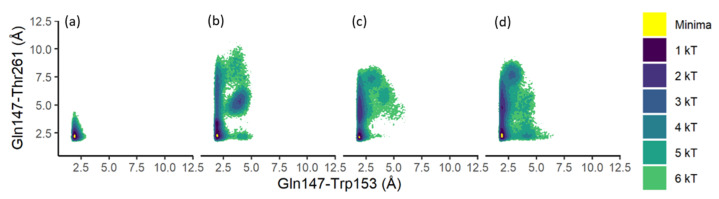
ΔG distribution of the paired Gln-Trp and Gln-Thr distances. (**a**) Substrate; (**b**) Product; (**c**) Apoprotein models; (**d**) CoA models. Values less than 0.1 kT shown in yellow represent minima.

For each simulation of each model, every observed paired distance between Gln147-Trp153 and Gln147-Thr261 was grouped into bins of tenths of angstroms (distribution by population for all models in Figures S2 and S3). The free energy difference between these conformations, ΔG, for the combined Substrate, Product, Apoprotein, and CoA models, is shown in Figure 2a–d, respectively (Table 1a–d). ΔG was calculated as −kT ln(ρi/ρmax), with ρi the population of bin i, and ρmax the population of the most populated bin. Bins without observations were given an arbitrary ΔG value of 10 kT. Individual simulation free energy plots appear in Figure S4.

For the combined Substrate model simulations, the ΔG minimum appeared at Gln147-Trp153 = 2.0 Å and Gln147-Thr261 = 2.2 Å and included 3.6% of total observations, whereas the combined Product model simulations had a ΔG minimum of Gln147-Trp153 = 2.0 Å and Gln147-Thr261 = 2.3 Å, and included 1.2% of the total observations. Table 2 presents the percentage of observations for each group in order of increasing ΔG kT levels for each combined model simulation. While the ΔG minima were similar, the Product model simulations revealed a greater fluctuation in the distances between the two hydrogen bonding pairs; the combined Product model simulations indicate that the Gln147-Thr261 distance varies from the ΔG minimum distance of 2.3 Å to approximately 7.5 Å along a 3 kT pathway, i.e., over 3 times more than that observed in the combined Substrate models. The distance between Gln147 and Trp153 also varies, with a subpopulation at approximately Gln147-Trp153 = 4.1 Å and Gln147-Thr261 = 5.6 Å. This subpopulation of 2 kT, 3 kT, and 4 kT includes 24.5% of the total combined Product model simulations (individual percent by kT value of 2.1%, 14.8%, and 7.6%, respectively). Taken together, while the Substrate models maintained the close proximity of the three residues of interest, the Product models demonstrated greater variation in distances, including a secondary energetically favorable conformation wherein the three residues exhibited more than a two times greater distance than those observed in the Substrate models.

The combined Apoprotein model simulations (Table 1c) displayed a ΔG minimum at Gln147-Trp153 = 1.9 Å and Gln147-Thr261 = 2.2 Å and included 1.5% of total observations. These simulation results demonstrated a variation in the distance between the Gln147-Thr261 residues. For example, a 2 kT subpopulation centered at Gln147-Trp153 = 2.0 Å and Gln147-Thr261 = 4.3 Å includes 25.8% of total observations and was encompassed by a 3 kT landscape that also included the ΔG minimum observations. The combined CoA model simulations revealed the ΔG minimum at Gln147-Trp153 = 2.0 Å and Gln147-Thr261 = 2.3 Å, and included 1.1% of total observations. The distribution of observations was similar to those observed for the combined Apoprotein models, but with a lower energetic barrier. While the combined Apoprotein model simulations demonstrated a 2 kT subpopulation at Gln147-Trp153 = 2.0 Å and Gln147-Thr261 = 4.3 Å, the CoA models exhibited a 1 kT subpopulation at Gln147-Trp153 = 1.9 Å and Gln147-Thr261 = 4.9 Å, accounting for 2.5% of observations. This 1 kT subpopulation was surrounded by a 2 kT subpopulation that also surrounded the CoA ΔG minimum observations. The combined CoA models also exhibited a small subpopulation of observations (2.6% of the total) at more than 3.5 times the distance between Gln147-Thr261, 7.5 Å, of those observed in the combined Substrate models. Taken together, the Apoprotein and CoA findings indicate that while the Apoprotein frequently stabilizes a low distance Gln147-Thr261 interaction in the Substrate model, the CoA headgroup stabilizes a higher Gln147-Thr261 distance. Findings from the present work suggest a highly dynamic role of the three residues of interest, with a structure that responds to the presence of a ligand of the CoA headgroup, of a saturated substrate, and of a desaturated product.

### 3.3. Substrate Tunnel Kink and C9-C10 Dihedral

**Figure 3:**
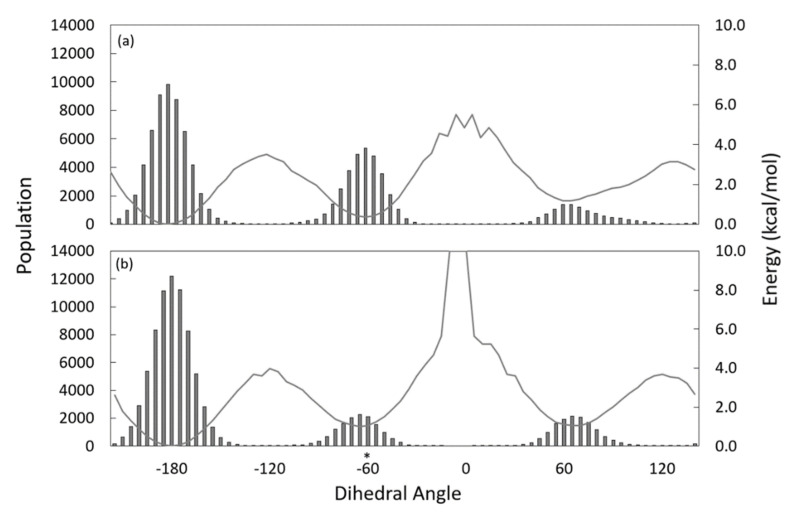
Distribution of the dihedral angle of the stearoyl-CoA formed by the 8th, 9th, 10th and 11th carbon atoms with corresponding free energy. Results for the five simulations grouped by (**a**) substrate when in the protein; (**b**) substrate when in waterbox. Asterix indicates the −60° dihedral angle that favors the desaturation reaction.

The crystallographically observed kink of the Substrate model (Table 1a) contributed to a favorable positioning of the substrate in the tunnel. SCD1 desaturated at C9-C10 of the lipid tail of the stearoyl-CoA substrate. A negative gauche dihedral about C9-C10, rather than a trans conformation, was proposed as the favored conformation for the desaturation reaction [2]. Figure S5 shows ρ_(__χ)_, the distribution of the χ dihedral angle of the stearoyl-CoA formed by the 8th, 9th, 10th, and 11th carbon atoms in each of the five Substrate simulations and the five Substrate-waterbox simulations, and Figure 3 shows these results grouped by condition (i.e., Substrate vs. Substrate-waterbox). The free energy corresponding to rotations around this dihedral, calculated as ϕ = −0.6 ln (ρ_(χ)_) (in kcal/mol), is represented by the overlaid gray line. The actionable dihedral, −60°, is indicated with an asterisk. The dihedral distribution in each of the five Substrate-waterbox simulations suggests that when the substrate is in a waterbox rather than the SCD1 substrate tunnel, the predominant substrate conformation is trans. The dihedral distribution observed in the Substrate model, i.e., with the saturated C9-C10 bond in the protein, shows a nearly equal population of trans and of negative gauche dihedral conformations in Substrate simulations 1, 3, and 5, each with the greatest population of observations within 5 degrees of the −60 dihedral. However, the trans conformation was favored in Substrate simulations 2 and 4. Comparison of these models suggests that the protein environment of the substrate catalyzes the population of an actionable conformation for the C9-C10 bond.

### 3.4. Residue Distance and Tunnel Shape

**Figure 4:**
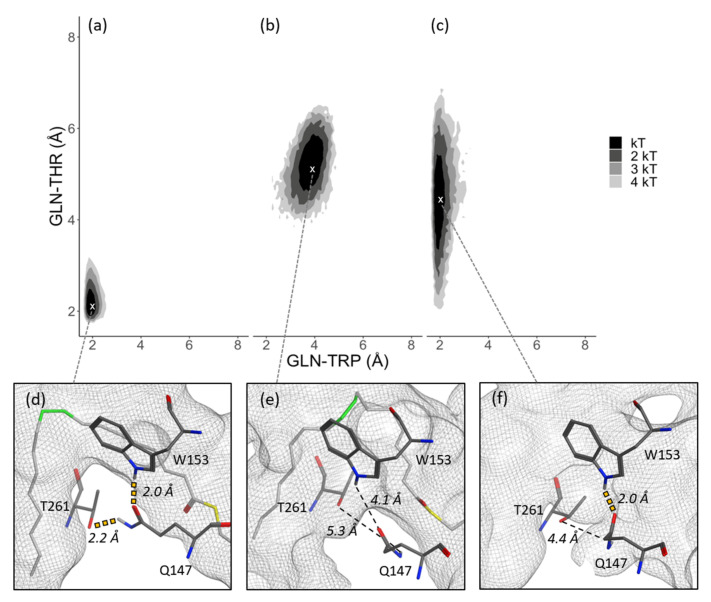
ΔG distribution of the paired Gln-Trp and Gln-Thr distances. (**a**) Substrate simulation 1, (**b**) Product simulation 1, (**c**) Apoprotein simulation 1 models. Minima indicated with white “x”. (**d**–**f**) Binding tunnel (gray mesh) for each corresponding ΔG minima of a, b, and c, with distances between Gln-Trp and Gln-Thr within hydrogen bonding range (yellow dotted line), and outside of hydrogen bonding range (black dashed line).

Hydrogen bond interactions between Gln147-Trp153 and Gln147-Thr261 have been suggested to contribute to the kink in the substrate binding tunnel [19]. The results from the present work provide partial support for this attribution. Figure 4 presents a comparison of the free energy plots of the Substrate simulation 1 model, Product simulation 1 model, and Apoprotein simulation 1 model (Figure 4a–c), shown with corresponding figures of the geometry of the binding sites (Figure 4d–f). In the Substrate simulation 1 model (Table 1a), the gray mesh surface of the tunnel is in the crystallographic kinked geometry (Figure 1b), whereas there is no such kink in the Product simulation 1 model (Table 1b), as shown by the comparatively more spacious gray mesh tunnel (Figure 4e). Taken together, the three residues are closer together in the Substrate simulation 1 model (Table 1a), and are observed within a kinked tunnel, whereas the three residues are farther apart in the Product simulation 1 model (Table 1b), which is not kinked. The distribution of Gln147-Thr261 and Gln147-Trp153 distances in the Apoprotein simulation 1 model (Table 1c) exhibits distributions that are in between those of the Substrate simulation 1 and the Product simulation 1. The trajectory of these distances, shown in Figures S2 and S3, respectively, indicates that the Gln147-Trp153 distance remained more consistently close compared to the Gln147-Thr261 distance, which fluctuated. All five Substrate simulations revealed this pattern, which supports the hypothesis that hydrogen bond interactions between Gln147-Trp153 and Gln147-Thr261 correspond to the kink in the substrate binding tunnel. Furthermore, results from the combined Substrate simulations, when the three residues were within hydrogen bonding range (i.e., >2.2 Å), showed that 18.1% of dihedrals were within five degrees of negative gauche, whereas this negative gauche conformation was observed for only 3.5% of dihedrals when the three residues were not within hydrogen bonding range. The stearoyl-CoA C11-C18 lipid tail extended into the binding tunnel in a similar manner when the C9-C10 dihedral was in negative gauche or trans position (Figure S6), indicating that the positioning of the tail can adapt to the shape of the tunnel through variations of lipid tail dihedral angles.

## 4. Discussion

**Scheme 1:**
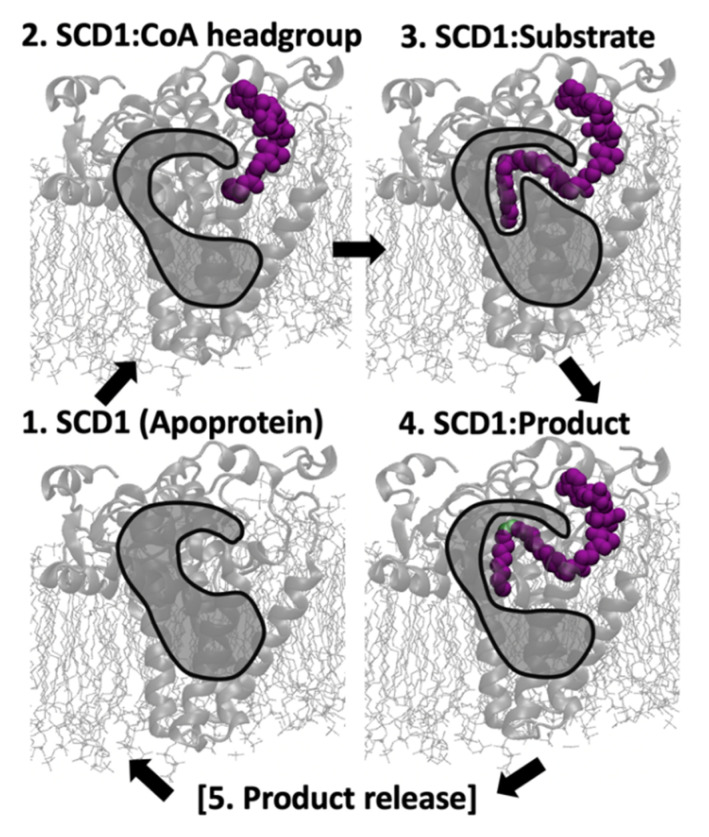
Proposed sequence of binding events. SCD1 appears in the membrane, with the hydrophobic tunnel represented by an opaque gray shape with black outline with purple ligand, desaturated carbons in green.

These results provide insight into the mechanism of SCD1’s interactions, from the apoprotein state to the initial contact of the substrate’s CoA moiety, to the positioning of the substrate in the tunnel, to the discrimination between the substrate and the product. Scheme 1 illustrates this proposed series of events: (1) The apoprotein exhibits fluctuations in distances between the two residues that connect one side of the tunnel to the other, Gln147-Thr261, resulting in a periodically less-restricted tunnel entrance; (2) CoA-moiety interacts with the protein surface, which further increases the favorability of greater distances between Gln147 and Thr261; (3) After the substrate inserts in the protein, the three residues remain consistently within hydrogen bonding range (i.e., kinked tunnel), a finding which corresponds to a higher proportion of favorable negative gauche substrate conformations; (4) After the substrate is catalyzed into the product, the distances between both Gln147-Trp153 and Gln147-Thr261 are more likely to be observed at greater distances, possibly allowing for egress of the product into the membrane; and (5) Product release indicates the presumed transition from the Product model to the Apoprotein model, which was not simulated in the present work.

### 4.1. From Apoprotein to Substrate Insertion

Based on their crystal structure observations, Wang et al. [18] hypothesized that the order of interactions between the stearoyl-CoA substrate and the apoprotein begin with the interaction of the CoA moiety and the nine protein face residues, followed by the movement of the fatty acid tail proceeding towards the tunnel entrance, and passing between TM2 and TM4. The present results are largely consistent with this view. The three residues of interest have higher energetic penalties when they are more distant in the Apoprotein model than the CoA model (Table 1c,d, respectively). Because the increased distance between Gln147 and Thr261 was observed in the CoA model (Table 1f) but not in the Apoprotein model (Table 1c), the tunnel-opening action of CoA appears to be due to an allosteric change based on the interaction between these nine residues and the CoA moiety. This finding is consistent with experimental results that indicate that SCD1 acts on the stearoyl-CoA but not the stearate, which is the saturated lipid tail without the CoA headgroup [2]. Given these findings, the series of actions leading to stearoyl-CoA entry into SCD1 may be as follows (see Scheme 1, steps 1 and 2): the residues that the CoA moiety interact with serve as a “doorbell” for the CoA head group of the incoming substrate to press.

Mutagenesis studies of the membrane desaturase rat D6D, which does not presently have a determined structure, indicated eight tunnel residues that contribute to substrate specificity, including Trp, Gln, and Ser residues [26]. As these residues contribute to substrate specificity, they have been proposed to be near the binding tunnel entrance. However, due to the low identity and differing substrates between rat D6D and human D9D, it is not possible to assert whether or not the rat D6D’s Trp, Gln, and Ser residues are equivalent, structurally and functionally, to the Trp, Gln, and Thr described in the present work on human D9D.

### 4.2. From Inserted Substrate to Product

The combined Substrate model (Table 1a) free energy distribution indicates that when the two paired distances, Gln147-Trp153 and Gln147-Thr261, were simultaneously close, the dihedral about the stearoyl-CoA C9-C10 bond was more likely to be actionable (−60° ± 10°) than when they were further apart. These results contrast with those of the stearoyl-CoA in the Substrate-waterbox model, which consistently stabilized the trans conformation over the negative gauche conformation (Figure 3 and Figure S5). In this way, SCD1 stabilizes the substrate for desaturation. Consistent with the close proximity of Gln147-Trp153 and Gln147-Thr261 observed in the human and murine crystal structures, the Substrate model (Table 1a) results indicate that the most energetically favorable conformation occurred when the residues were within hydrogen bonding distance. On the other hand, the Product model exhibited larger distances between these residues at the ΔG distribution minimum. This geometry, inconsistent with hydrogen bonding, corresponds to an open tunnel shape. Taken together, the substrate:protein and product:protein interactions are substantially different, suggesting a route for substrate-product discrimination related to the positions, Trp153-Gln147-Thr261. (Scheme 1, Steps 3 and 4)

## 5. Conclusions

The current results describe the dynamics of SCD1 in the apoprotein state, with an interacting CoA headgroup, with bound substrate, and with bound product. This work describes a structure–function relationship characterization of D9D. These findings assist in prioritizing experimentally directed mutagenesis work. A sequence of dynamic events drives the protein:substrate recognition and processing and the release of the substrate. As summarized in Scheme 1, results indicate that, (1) the Apoprotein has a shallow entrance to the tunnel, (2) the CoA headgroup opens the tunnel entrance, (3) hydrogen bonding between Gln147-Trp153 and Gln147-Thr261 contributes to the tunnel kink typical of the Substrate model, and (4) the distance between these three residues in the Product model may aid in product release into the membrane. Furthermore, the CoA headgroup may aid in substrate–product discrimination.

## Figures and Tables

**Table 1:** Model names and descriptions.

Name	Description	Ligand Figure
a. Substrate	Stearoyl-CoA with SCD1	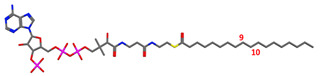
b. Product	Oleoyl-CoA with SCD1	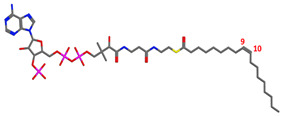
c. Apoprotein	SCD1	*NA*
d. CoA	CoA moiety with SCD1	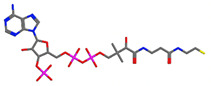
e. Substrate-waterbox	Stearoyl-CoA in waterbox (no membrane or protein)	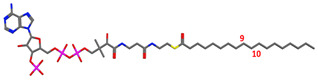

**Table 2:** Percentage of paired Gln147-Thr261 and Gln147-Trp153 distances for each model grouped by kT.

kT	Substrate	Product
Minimum	3.6	1.2
1 kT: <0.6	54.0	31.4
2 kT: 0.6–1.19	25.1	17.8
3 kT: 1.2–1.79	9.3	23.5
4 kT: 1.8–2.39	4.6	12.9
5 kT: 2.4–2.99	1.7	6.8
6 kT: 3.0–3.59	0.9	5.1
7 kT: 3.6–4.19	0.4	0.7
8 kT: 4.2–4.79	0.2	0.6
9 kT: 4.8–5.39	0.2	0.0

## Data Availability

Model files may be found in the Zenodo repository at https://zenodo.org/record/4245236.

## Supplementary Materials

The following are available online at https://www.mdpi.com/article/10.3390/biom11101435/s1: Figure S1: Distribution of iron distances for combined Substrate model simulations; Figure S2: Distance between hydrogen bonding partners Gln147 and Thr261 across 100-ns simulations; Figure S3: Distance between hydrogen bonding partners Gln147 and Trp153 across 100-ns simulations; Figure S4: ΔG distribution of the paired Gln-Trp and Gln-Thr distances for each kT level; Figure S5: Individual waterbox simulations compared to substrate simulations; Figure S6: Comparison of stearoyl-CoA lipid tails when the dihedral about C9 and C10 is in negative gauche (coral) vs. trans (gold) position; Table S1: Mean hydrogen bond distance and percent putative hydrogen bonding over 100-ns trajectory.
