# Supplementary material for: Sequential Dynamics of Stearoyl-CoA Desaturase-1(SCD1)/Ligand Binding and Unbinding Mechanism: A Computational Study
Source: Biomolecules. 2021 Sep 30;11(10):1435. doi: 10.3390/biom11101435 (PMC8533217; doi:10.3390/biom11101435)
Supplement: Supplementary file 1 [file biomolecules-11-01435-s001.zip › biomolecules-1379892-supplementary.pdf]

Sequential Dynamics of Stearoyl-CoA Desaturase-1(SCD1)/Ligand Binding and Unbinding Mechanism: A Computational Study

SI Material: Figures S1–S6 and Table S1

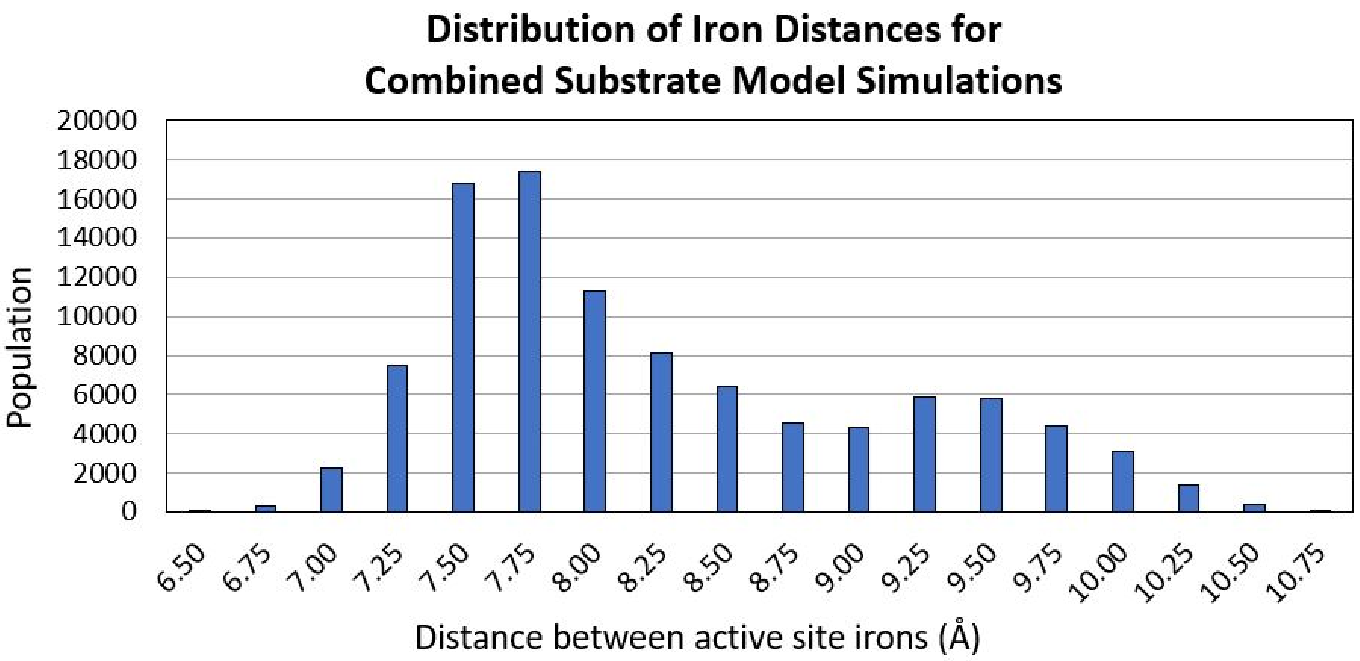

Figure S1. Distribution of iron distances for combined Substrate model simulations. Asterix indicates distance identified in SCD1 crystal structure.

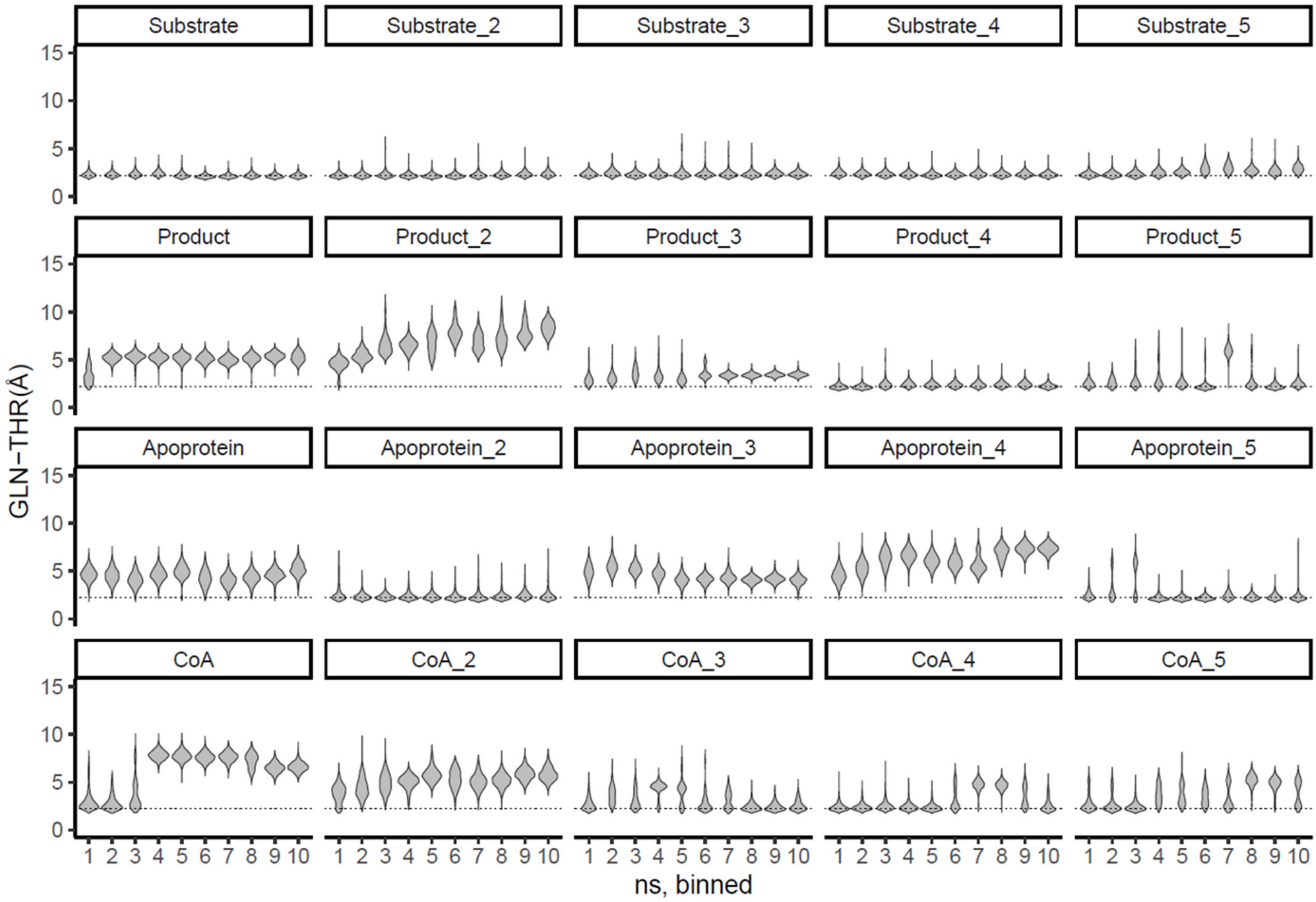

Figure S2. Distance between hydrogen bonding partners Gln147 and Thr261 across 100-ns simulation. Dashed line indicates maximum hydrogen bonding distance (2.2 Å).

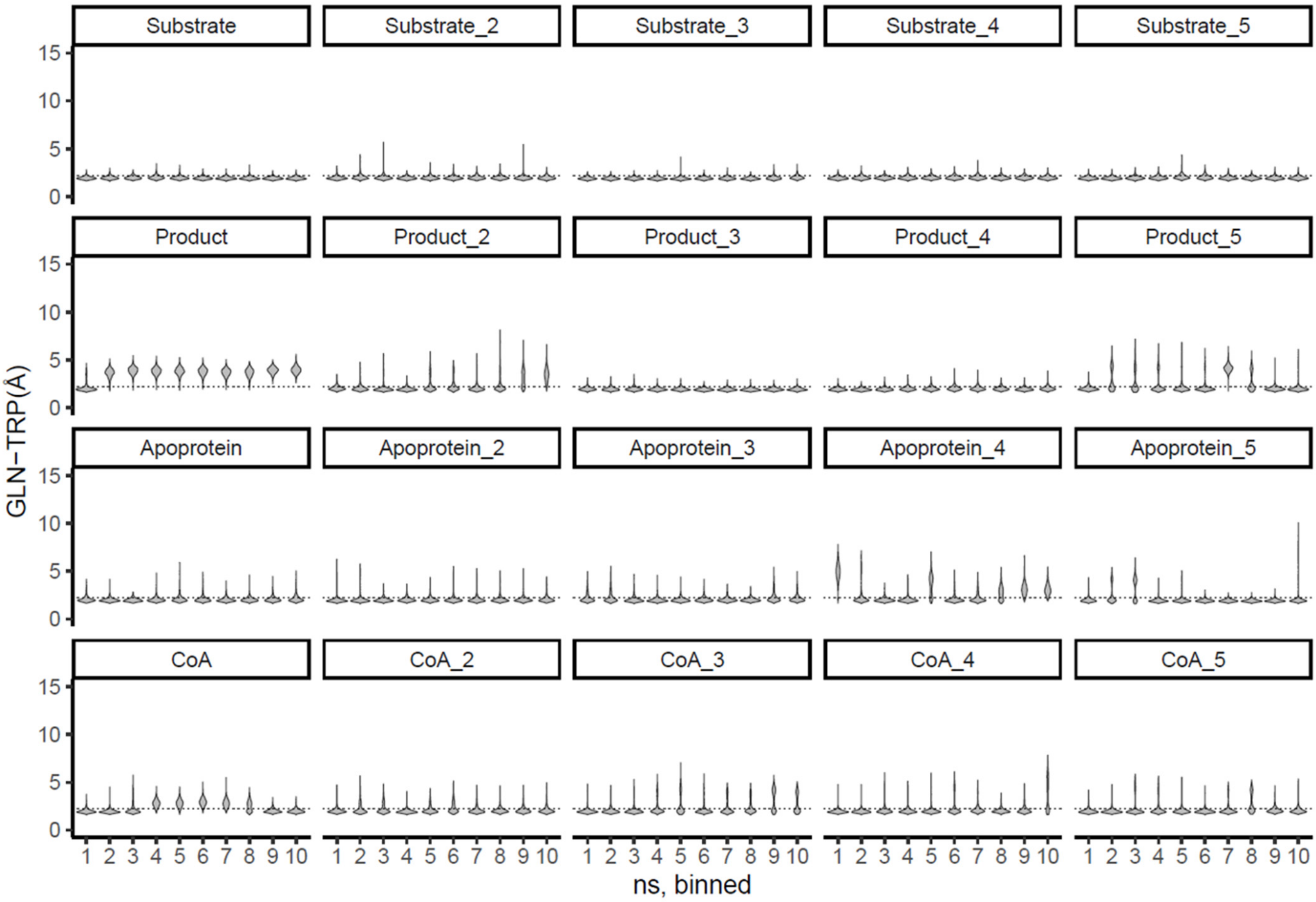

Figure S3. Distance between hydrogen bonding partners Gln147 and Trp153 across 100-ns simulation. Dashed line indicates maximum hydrogen bonding distance (2.2 Å).

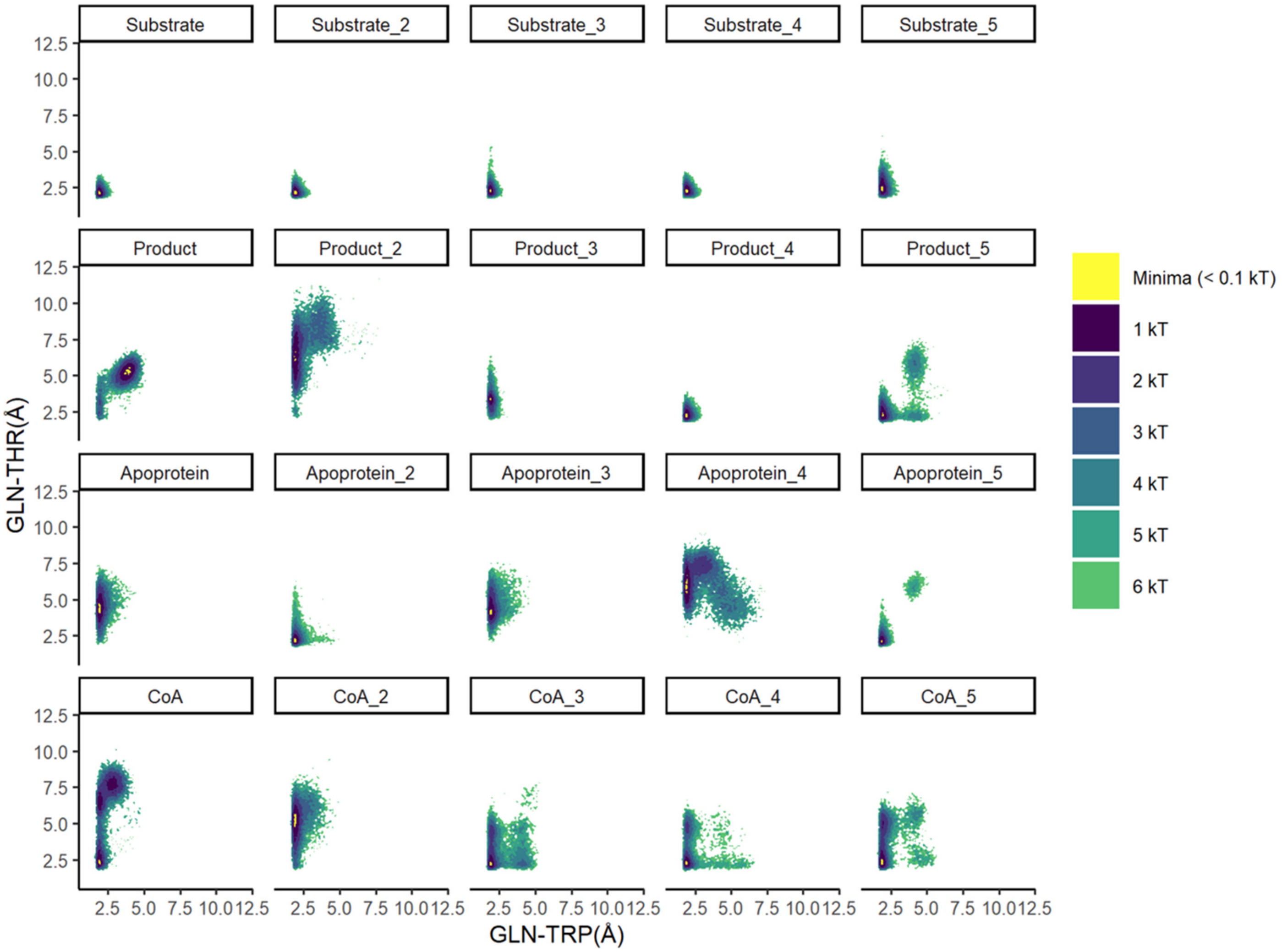

Figure S4. ΔG distribution of the paired Gln-Trp and Gln-Thr distances for each kT level.

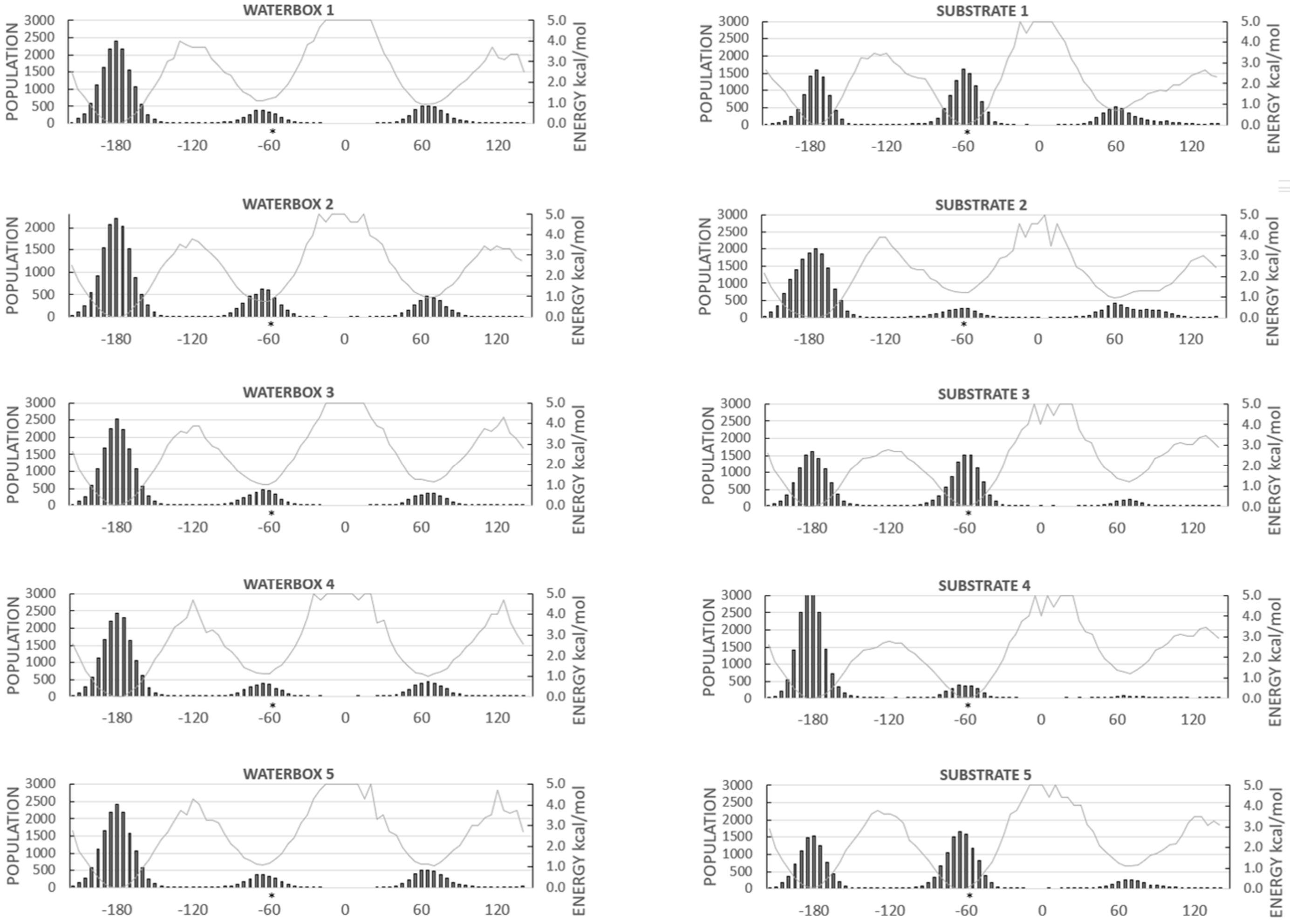

Figure S5. Individual waterbox simulations compared to substrate simulations. Distribution of the dihedral angle of the stearoyl-CoA formed by the 8th, 9th, 10th and 11th carbon atoms with corresponding free energy. Asterix indicates the −60° dihedral angle that favors the desaturation reaction.

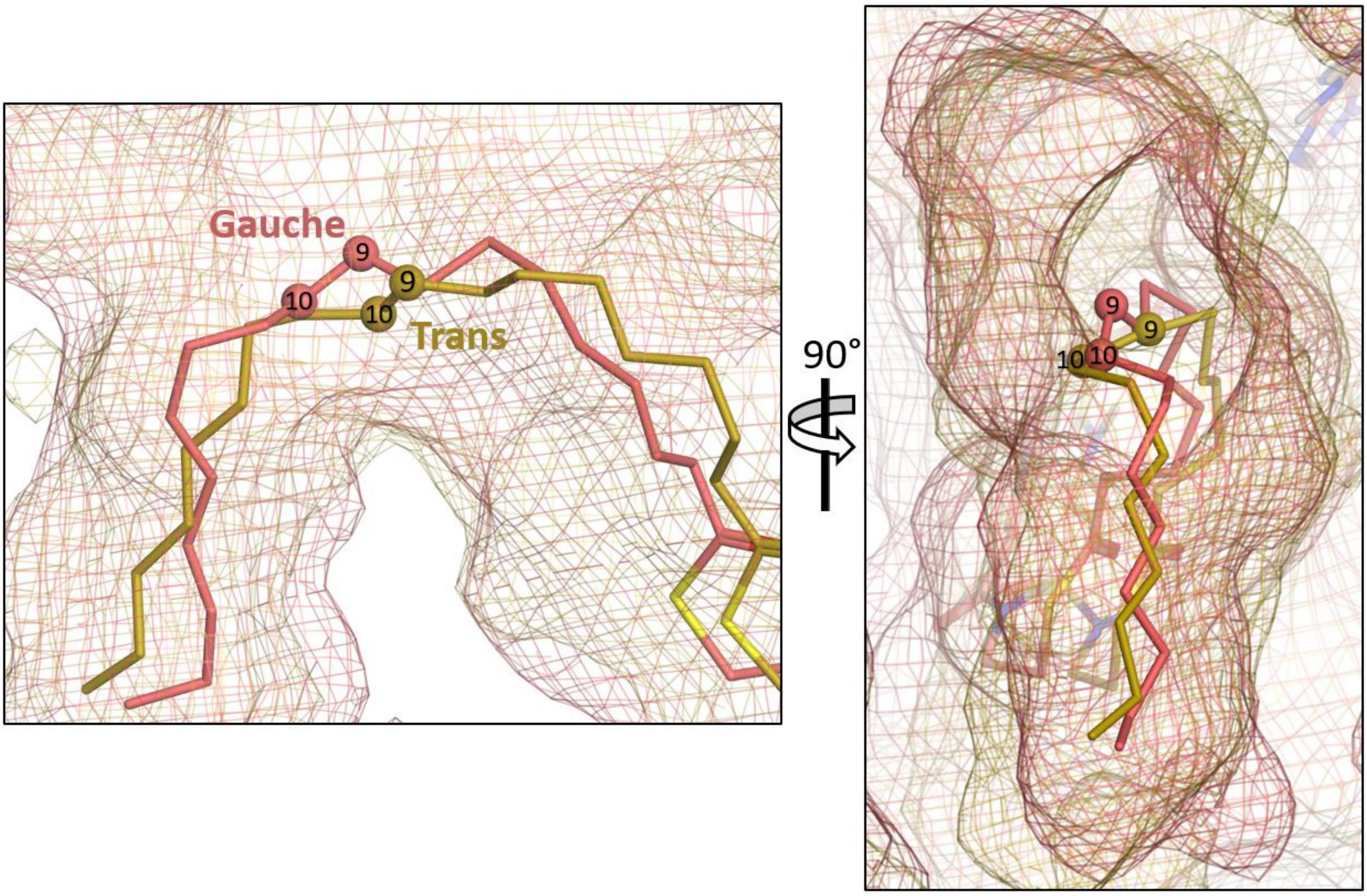

Figure S6. Comparison of stearoyl-CoA lipid tail when the dihedral about C9 and C10 is in negative gauche (coral) vs. trans (gold) position. The tunnel shape is shown as a mesh in the corresponding colors. C9 and C10 are indicated as balls, whereas the rest of the stearoyl-CoA is shown as lines. The overlaid images are example frames from the Substrate model trajectory.

Table S1. Mean hydrogen bond distance and percent putative hydrogen bonding over 100-ns trajectory. "Both%" column indicates the proportion of observations in which both Gln147-Trp153 and Gln147-Thr261 exhibit simultaneous hydrogen bonding (2.2 Å).

**Condition**	**Residue Pairs**						
	Gln147-Trp153			Gln147-Thr261			Both
	mean	sd	%	mean	sd	%	%
Substrate models							
Substrate 1	2.0	0.2	91.3	2.3	0.3	49.3	45.0
Substrate 2	2.0	0.2	88.1	2.3	0.3	46.6	40.4
Substrate 3	1.9	0.2	94.0	2.4	0.4	30.3	28.0
Substrate 4	2.0	0.2	87.8	2.4	0.3	31.5	26.5
Substrate 5	2.0	0.2	88.4	2.6	0.5	15.9	13.4
Average	2.0	0.2	89.9	2.4	0.3	34.7	30.7
Product models							
Product 1	3.6	0.7	8.2	5.0	0.8	0.5	0.4
Product 2	2.4	0.8	61.9	6.8	1.5	0.0	0.0
Product 3	2.0	0.2	92.3	3.3	0.6	1.1	0.8
Product 4	2.0	0.2	87.1	2.4	0.3	32.2	28.1
Product 5	2.6	1.0	62.7	3.0	1.3	23.2	16.0
Average	2.5	0.6	62.4	4.1	0.9	11.4	9.1
Apoprotein models							
Apoprotein 1	2.1	0.3	80.4	4.5	0.8	0.3	0.2
Apoprotein 2	2.1	0.4	83.3	2.4	0.6	37.5	32.2
Apoprotein 3	2.1	0.4	77.3	4.5	0.8	0.0	0.0
Apoprotein 4	2.8	1.1	44.7	6.2	1.1	0.0	0.0
Apoprotein 5	2.2	0.7	84.6	2.6	1.1	41.4	39.0
Average	2.2	0.6	74.1	4.1	0.9	15.8	14.3
CoA models							
CoA 1	2.4	0.6	47.8	6.1	2.1	3.5	3.1
CoA 2	2.1	0.4	73.9	5.1	1.1	0.7	0.6
CoA 3	2.4	0.8	67.3	3.1	1.1	18.1	12.6
CoA 4	2.3	0.8	75.4	3.1	1.1	20.5	15.1
CoA 5	2.2	0.7	76.3	3.7	1.3	10.8	9.4
Average	2.3	0.7	68.1	4.2	1.3	10.7	8.2
